# Enhancing Red Yeast Biomass Yield and Lipid Biosynthesis by Using Waste Nitrogen Source by Glucose Fed-Batch at Low Temperature

**DOI:** 10.3390/microorganisms10061253

**Published:** 2022-06-20

**Authors:** Iwona Gientka, Magdalena Wirkowska-Wojdyła, Ewa Ostrowska-Ligęza, Monika Janowicz, Lidia Reczek, Alicja Synowiec, Stanisław Błażejak

**Affiliations:** 1 Department of Food Biotechnology and Microbiology, Institute of Food Science, Warsaw University of Life Sciences-SGGW, Nowoursynowska Str. 159c, 02-776 Warsaw, Poland; alicja_synowiec@sggw.edu.pl (A.S.); stanislaw_blazejak@sggw.edu.pl (S.B.); 2 Department of Chemistry, Institute of Food Sciences, Warsaw University of Life Sciences-SGGW, Nowoursynowska Str. 166, 02-787 Warsaw, Poland; magdalena_wirkowska@sggw.edu.pl (M.W.-W.); ewa_ostrowska_ligeza@sggw.edu.pl (E.O.-L.); 3 Department of Food Engineering and Process Management, Institute of Food Science, Warsaw University of Life Sciences-SGGW, Nowoursynowska Str. 159c, 02-776 Warsaw, Poland; monika_janowicz@sggw.edu.pl; 4 Institute of Environmental Engineering, Warsaw University of Life Sciences–SGGW, Nowoursynowska Str. 166, 02-787 Warsaw, Poland; lidia_reczek@sggw.edu.pl

**Keywords:** *Rhodotorula*, fed-batch, lipid biosynthesis, SCO, PDSC, low temperature, fatty acids, oleic acid

## Abstract

This work reports the effect of simple feeding strategies and temperature to obtain high-cell-density cultures of *Rhodotorula glutinis* var. *rubescens LOCKR13* maximizing the de novo lipid productivity using deproteinated potato wastewater (DPW) as a basic medium. Feeding DPW with glucose enables a high yield of *Rhodotorula glutinis* var. *rubescens* *LOCKR13* biomass (52 g _d.w._ L^−1^) to be obtained. The highest values of lipid accumulation (34.15%, *w/w*), production (14.68 g L^−1^) and yield coefficients (Y_L/S_: 0.242 g g^−1^), and volumetric productivity (P_L_: 0.1 g L^−1^ h^−1^) were reached by the strain in the two-stage fed-batch process at 20 °C. The lipid of yeast biomass was rich in oleic acid (^Δ9^C18:1) and palmitic acid (C16:0), and the lower temperature of incubation significantly increased the MUFA (especially oleic acid) content. For the first time, a unique set of thermal analyses of the microbial oil was performed. The isotherms of the oxidation kinetics (PDSC) showed that lipids extracted from the biomass of red yeast had high oxidative stability. This feature of the yeast oil can be useful for long-shelf-life food products and can be promising for the production of biodiesel.

## 1. Introduction

Single-cell oils (SCO), depending on the composition of fatty acids, can be used as food, food additives such as infant formula milk, and feed. Microbial fat can also be used as a substrate for biodiesel production [1]. The typical oleaginous yeast genera (able to accumulate lipids of more than 20% of their cellular dry weight) include *Apiotrichum, Candida, Cryptococcus, Lipomyces, Metschnikowia, Trichosporon, Yarrowia,* and some red yeast such as *Rhodotorula* or *Rhodosporidium* [2,3,4]. The red, pink, or orange colonies as a consequence of yeast’s ability for carotenoid biosynthesis are also characteristic of yeasts belonging to *Phaffia* and *Sporobolomyces* [5]. The *Rhodotorula* red yeast can simultaneously produce lipids and carotenoids [6], but also exoglycolipids and β-glucans [7], multiplying the biotechnological usefulness of this genera. Microbial oils may have different physicochemical properties, which determines their usage with applications not only for nutrition but also for fuels. The large content of unsaturated and monounsaturated acids in microbial oil mainly determines its biodiesel properties [8], whereas the food, feed, cosmetic, and pharmaceutical industry are interested in microbial oils, which could be an alternative for cocoa butter or shea butter, as well as being rich in PUFA or a low-cost source of specific fatty acids such as DHA or EPA.

Temperature influences various life processes such as cell growth, energy production, metabolic activity, and significantly the biosynthesis of intracellular compounds. For adaptation to low temperatures, cells can modulate membrane fluidity [9]. The membrane fluidity is dependent on the compositions of fatty acids in membrane lipids [10,11], and the increase in polyunsaturated fatty acids (PUFAs) content can reduce their melting point.

Two main barriers to industrial SCO production are the relatively low yield per unit volume of the medium and the cost-effectiveness of the process, where the latter is significantly determined by the cost of the substrate. It is well known that de novo lipid biosynthesis occurs when the cells cope with nitrogen depletion with an excess carbon source [12]. Glucose is a basic and commonly supplied carbohydrate in the media used for lipid biosynthesis in yeast [2]. Some attempts have been made to reduce the costs of fungal SCO production by selecting low-cost sources of carbon in media. They could be the hydrophilic industrial byproducts or wastewaters from the food industry such as corn syrups [13], hydrolysate of cassava starch [14], whey [15], or monosodium glutamate wastewater [16]. However, ex novo lipid synthesis requires low-cost hydrophobic substrates derived from the fishery industry, olives production [17], as well as waste cooking oils [18]. In addition, wheat bran and cornmeal with animal fats addition were used recently in a solid-state state fermentation matrix for oleaginous fungi [19]. To increase the profitability of microbial lipids production, nonaseptic technologies have started to develop [17]. According to the fixed capital investment, cost of operating labor, raw materials, and waste treatment, the total cost of 1 kg of microbial oil in 2014 was USD 5.48 for a price of glucose of USD 400/t [20]. In 2022, the approximate price range for glucose is 40–45% higher [21], as well as energy, water, and other media. To lower the cost of SCOs, research must be applied in designing products where the whole cell is used in continuous processing, and an effective biorefinery must be created [22].

Deproteinated potato wastewater (DPW) is a liquid waste of the starch industry (over 12 million m^3^/year) [8], which can be a source of nitrogen in the media used for yeast biomass growth and the biosynthesis of microbial oils [23]. The high productivity depends not only on the possibilities of the strain to accumulate lipids in lipid bodies but also on biomass yield [20]. 

The fed-batch process, which is also known as a semi-batch culture, can be performed intermittently or continuously, adding to the culture vessel the nutrients necessary for cell growth or product formation during the operation [24,25]. Consequently, the concentration of limiting substances that provides nourishment essential for growth can be maintained at an optimal level by adjusting the feed rates. This strategy can maximize the product yield at the end of the cultivation [25]. During the industrial process of lipid production, the high-yeast-cell-density cultures are the basis. One factor that limits the growth of the aerobic yeast and consequently cell density in flask cultures is the high oxygen demand. Therefore, a high yeast cell density can only be achieved in bioreactors with efficient aeration. A high density of oleaginous cell cultures has been achieved for *R. toruloides* and *C. curvatus* using fed-batch [26].

All lipids, including microbiological ones, are susceptible to unfavorable changes, and the main cause of their degradation is oxidation. The number of publications characterizing the oxidative stability of microbial fats is negligible.

The effect of glucose feeding in media with a waste nitrogen source on oleaginous yeast cells at different temperatures has not been studied so far. Our goal was to understand the metabolic response of *Rhodotorula glutinis* var*. rubescens LOCKR13* yeast cells during bioreactor culture in deproteinated potato wastewater fed with glucose and at different temperatures, as well as determine the oxidation susceptibility of accumulated intracellular lipids based on the use of differential scanning calorimetry.

## 2. Materials and Methods

### 2.1. Strain

The biological material was the yeast strain *Rhodotorula glutinis* var. *rubescens LOCKR13* originating from the Technical University of Lodz Culture Collection, Poland. 

### 2.2. Industrial Waste Used as Component of the Medium

The potato wastewater (DPW) was obtained after the deproteinization stage from the technological line of starch production (PEPEES S.A., Łomża, Poland), transported to the laboratory, and immediately sterilized in an autoclave (121 °C/0.1 MPa/20 min) to preserve for storage (HiCLAVE HG-80 autoclave, HMC Europe). To remove all precipitates formed during sterilization, DPW was centrifuged at 3200× *g* for 20 min (Eppendorf 5810 Centrifuge). The used carbon source was glucose at an initial concentration of 50 g per 1 L of DPW, the pH was adjusted to 5.6, and then the medium was sterilized (121 °C/0.1 MPa/20 min) (HiCLAVE HG-80 autoclave, HMC Europe). 

### 2.3. Inoculation, Batch Culture, and Fed-Batch Culture Conditions

YPD with a pH 5.6 medium was used for inoculation culture. The inoculating cultures (100 mL of YPD medium in flasks of 500 mL volume) were incubated for 48 h at 28 °C on a reciprocating shaker (SM-30 Control, Buechler, Bodelshausen, Germany) at a frequency of 200 cycles/min. Then, all cultivations (control-A and experimental–B and C) were carried out in a 5 L BioFlo 3000 bioreactor (New Brunswick Scientific Co., Edison, NJ, USA) with a working volume of 3 L. The growth temperatures were 20 °C and 28 °C, the agitation rate of the bioreactor was 200 rpm, and the air supply was maintained at a constant rate of 2 vvm. Formation was controlled by the addition of a silicone antifoaming agent (Sigma 204) to a final concentration of 2 mL L^−1^. 

In the one-stage feeding mode (experiment B), after the batch operation had run for 72 h, glucose (1:1, glucose:water solution) was fed into the bioreactor within a short period (approximately 15 min) to make the total concentration of glucose in the process 50 g L^−1^. In experiment C, the glucose solution was fed after the batch operation had run for 72 h, and 120 h up to the total concentration of 50 g L^−1^. The experiments were performed in duplicate. The culture broth samples (200 mL) were taken at 24 h intervals to determine the cell mass, lipids, and proteins concentration in biomass, glucose, and nitrogen residue. 

### 2.4. DPW Chemical Characteristics

In the first stage of the study, the DPW was characterized by determining the COD (by the dichromate method [27]) using Hach Lange cuvette tests (LCK014, HACH, Loveland, CO, USA) in the Water Centre of the Warsaw University of Life Sciences [28] and the total organic carbon content by the supercritical oxidation method (TOC Sievers Innovox, SUEZ, Paris, France) [29]. The content of reducing sugars (per glucose) was measured spectrophotometrically at λ = 550 nm using 3,5-dinitrosalicylic acid [30]. The nitrogen concentration was determined by the Kjeldahl method [31] using a KjelFlex K-360 (Büchi, Flawil, Switzerland) with the TitroLine 5000 automatic titrator (SIAnalytics, Weilheim, Germany). The protein content was determined by the Lowry method [32]. The waste was also determined for potassium (by FAES, flame atomic emission spectrometry), calcium, magnesium (FAAS), and phosphorus (ETAAS) in the Prof. Wacław Dąbrowski Institute of Agriculture and Food Biotechnology–State Research Institute (Warsaw, Poland). 

### 2.5. Biomass Yield and Lipids Content

The biomass yield (g _d.w_. L^−1^) was determined gravimetrically. Biomass samples for further analysis were centrifuged (6400× *g* for 20 min at 20 °C (Eppendorf 5810 Centrifuge)), twice-washed, and then lyophilized. First, the biomass was shook-cooled (Irinox MOD. 51 20 Italy) at an air temperature of 40 °C and stored under freezing conditions for 24 h in a CHRIST LCG Gamma 1-6 LSC lyophilizer (Martin Christ, Osterode am Harz, Germany) at 63 Pa [33] at a plate temperature of 20 °C. Lyophilized yeast biomass was crushed in a pulse mill (Pulverisette 0, Fritsch, Idar-Oberstein, Germany) for 5 min using a low range of pulsating. After lyophilization, the lipid content (% CDW) determination was performed by the gravimetrical method of Bligh and Dyer [34] with Zhang et al.’s [35] modification, which involves lipid extraction using chloroform and methanol. Briefly, to the sample of lyophilized and crushed yeast biomass (600 ± 30 mg), 5 mL of chloroform and 10 mL of methanol were added to the sample, and the mixture was vigorously stirred for ten minutes. Then, 5 mL of chloroform and 5 mL of water solution of NaCl (20%) were added and shaken again for 10 min. After centrifugation (6400× *g* for 10 min at 20 °C (Eppendorf 5810 Centrifuge)), the chloroform phase was transferred with a syringe to a previously weighed tube. The solvent was evaporated (Reacti-Vap III Evaporation Unit, Thermo Scientific, Waltham, MA, USA) under nitrogen [36] and gravimetrically determined overnight at 105 °C until a constant weight. The lipid titer (g L^−1^), also known as the volumetric lipid efficiency, was calculated as the amount of lipid extracted from the cells per liter of broth. The lipid content (% DCW) was defined as the percentage of lipid titer (g L^−1^) on the dry weight of biomass (g_d.w._ L^−1^).

### 2.6. Calculation of Bioprocess Efficiency Parameters

The weights of lipids, dry biomass, and glucose consumed were used to determine lipid/glucose (Y_L/S_), biomass/glucose (Y_X/S_), and lipid/biomass yield (Y_L/X_) coefficients, expressed as g g^−1^. The productivity of biomass (P_X_) and lipids (P_L_) was calculated by dividing their concentrations over time of the corresponding culture, expressed as g L^−1^ h^−1^. The efficiency of lipid production (ηL) as % of theoretical total lipid yield on the glucose was estimated by standard procedures.

### 2.7. Lipids Analysis-Relative Composition of Fatty Acids 

To determine the fatty acids content in extracted lipid samples, gas chromatography (GC) was used. According to [37], the fatty acids were converted into volatile methyl esters using a methanolic KOH solution and applied to the analytical column. The 30 m long BPX 70 capillary column (SGE Analytical Science, Milton Keynes, UK) with an inner diameter of 0.22 mm and a film thickness of 0.25 μm, and a YL6100 GC gas chromatography apparatus (Young Lin Bldg., Anyang, Hogye-dong, Korea) with a flame ionization detector were used. The oven temperature was programmed as follows: 60 °C for 5 min, increasing by 10 °C/min to 180 °C and from 180 to 230 °C at 3 °C/min. For another 15 min, the temperature was maintained at 230 °C. The temperature of the split injector was 225 °C, with a split ratio of 1:100. The detector temperature was maintained at 250 °C. Nitrogen (flow rate of 1 mL/min) was applied as a carrier gas. Fatty acid was identified by a comparison of their retention times with those of standard ones, quantified as a percent of the total of the FAMES content [38].

### 2.8. Thermal Analysis of Yeast Lipids 

The isotherms of the oxidation kinetics (PDSC Q 20 TA, TA Instruments, New Castle, DE, USA) were determined under isothermal conditions at a temperature of 120 °C and a pressure of 1400 kPa. The purge oxygen at a rate of 50 mL was used. The samples of 3–4 mg were placed in open aluminum pans and inserted into the heating chamber of the SCD cell. The reference pan was left empty [39]. Olive oil was examined as a control under the same conditions. The maximum oxidation temperature was determined as the maximum exothermal peak of oxidation.

### 2.9. Statistical Analysis

All tests were performed in duplicates. The results were analyzed using the statistical program homogeneity of variance and significance of differences between averages (two-way ANOVA analysis of variance and Tukey’s test, or one-way if it is written) at the significance level of α = 0.05 (TIBCO Statistic ver.13.3). The heatmap was obtained using the R platform. 

## 3. Results and Discussion

### 3.1. The Characteristic of the Potato Wastewater Used as a Medium

The chemical oxygen demand (COD) value of potato wastewater averaged 28.9 ± 1.87 g O_2_ L^−1^ (Table 1—last vers). The COD index is a significant parameter, which characterizes the level of waste purification. The high value of chemical oxygen demand of this waste showed the presence of a high content of both organic and inorganic compounds. Despite the industrial coagulation of proteins and further laboratory processing, the DPW used in this study was characterized by a high value of COD. However, the samples of waste reconstituted in laboratory processes directly from potatoes was 59 g O_2_ L^−1^ [40].

The average dry substance of DPW was 38.19 g L^−1^. The total organic carbon content was 11.716 ± 0.747 g L^−1^ and directly reducing sugars was 4.49 ± 0.44 g L^−1^. The potato wastewater was obtained after the thermal-acid deproteinization process from the technological line of starch production, then sterilized for preserving as well as for removing all participates such as residual starch and proteins. All those processes influence the chemical structure of waste, especially on the nitrogen content, which was 1.669 ± 0.047 g L^−1^, so the protein content calculated based on it was found to be 10.43 ± 0.5 g L^−1^. The protein concentration using the Lowry method was lower (3.48 ± 0.27 g L^−1^). The potassium content was 5.29 ± 0.9 g L^−1^ and was higher than those of nitrogen and phosphorus (0.665 ± 0.013 g L^−1^). Such a high concentration is a consequence of the naturally high concentration of this element in potato tuber. The contents of magnesium and calcium were 0.261 g L^−1^ and 0.014 g L^−1^.

Due to the different initial content of starch and sugars in potato varieties, as well as the technological parameters, a wide variety of reducing sugars, proteins, and phosphorus content in wastewater has been observed from year to year (Table 1). It may depend on the potato variety, degree of maturity, and weather conditions. In particular, the fluctuating protein content may result from the efficiency of the deproteinization process. In the case of waste used in this study, the juice water was obtained from the line after renovation. Such a procedure could increase the efficiency of machines and result in better protein separation. This hypothesis is also supported by the lower-than-usual values of COD and TOC. The deproteinized potato wastewater containing less than 1% of sugar cannot result in efficient cell growth and high biomass yield. Therefore, the DPW must be supplemented with an additional carbon source to best balance its availability in the media from this waste. In this study, glucose was used for this purpose.

### 3.2. Effect of Batch and Fed-Batch Strategy on Yeast Growth and Lipid Accumulation

**Figure 1:**
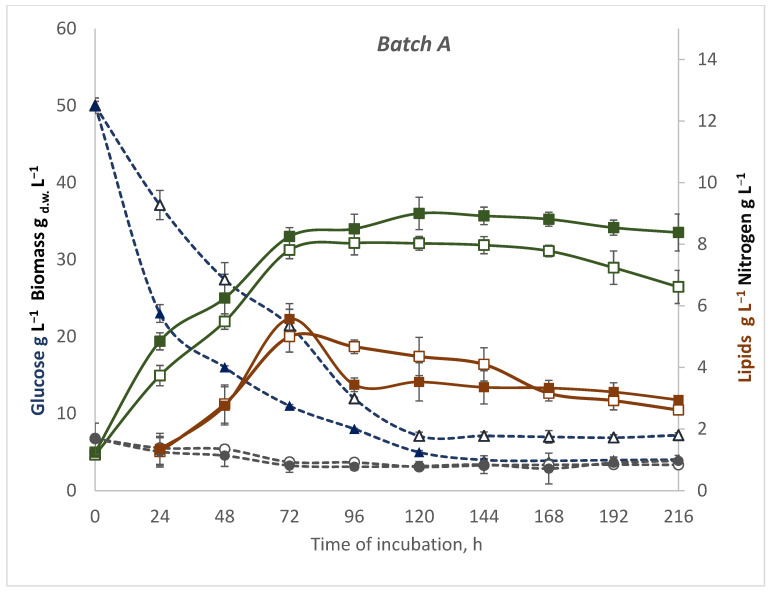
The timecourse of a batch A culture of *R. glutinis* var. *rubescens* in DPW and glucose medium containing initial concentrations of glucose 5% at two temperatures: 28 °C (filled tags) and 20 °C (empty tags). Kinetics of substrates: glucose—blue dotted line, nitrogen—grey dotted line, biomass—green line (where _d.w._—dry weight), lipids—brown line. Values represent the average of duplicate experiments.

Mechanisms of the effect of glucose feeding in media with a waste nitrogen source on the metabolism of yeast cells at different temperatures have not been studied so far. The course of growth curves (showed as biomass yield) and the rate of carbon and nitrogen sources and consumption depended on the cultivation strategy. Yeasts showed typical growth phases, and logarithmic and stationary phases in control batch (A) (Figure 1). The growth of yeast increased rapidly in the first days of incubation at 28 °C, reaching an average biomass yield of 36 g L^−1^ on the fifth day. However, during the next hours of cultivation, the cell mass did not increase. About 27 g L^−1^ of sugars was consumed by *R. glutinis* during the first 24 h of cultivation at 28 °C. Then, the sugar consumption rate decreased, such that about 7 g L^−1^ to 5 g L^−1^ of sugars was utilized per day on average before the 6th day of cultivation. From this moment to the last day of fermentation, the sugars consumed per day on average were further lower. Finally, the residual sugar concentration was 4.05 g L^−1^, and the total utilization of glucose was 91.9%. At lower temperature, the cell mass reached the maximum value of 32.15 g L^−1^ on the fourth day of cultivation. After one week of incubation, the biomass yield started to decrease. A significantly lower sugar consumption rate was observed, and during the first 24 h of incubation, it was only 12.97 g L^−1^ of sugars. In addition, the total degree of glucose utilization at a lower temperature of incubation was 86.2%. The curves of nitrogen utilization were similar regardless of temperature conditions. During control batch culture, the maximum total lipid was observed at the beginning of the stationary phase. The biomass contained up to 16% lipid at 20 °C and up to 16.89% lipid at 28 °C. The maximal efficiency of lipid production was 55% and 45%, respectively.

**Figure 2:**
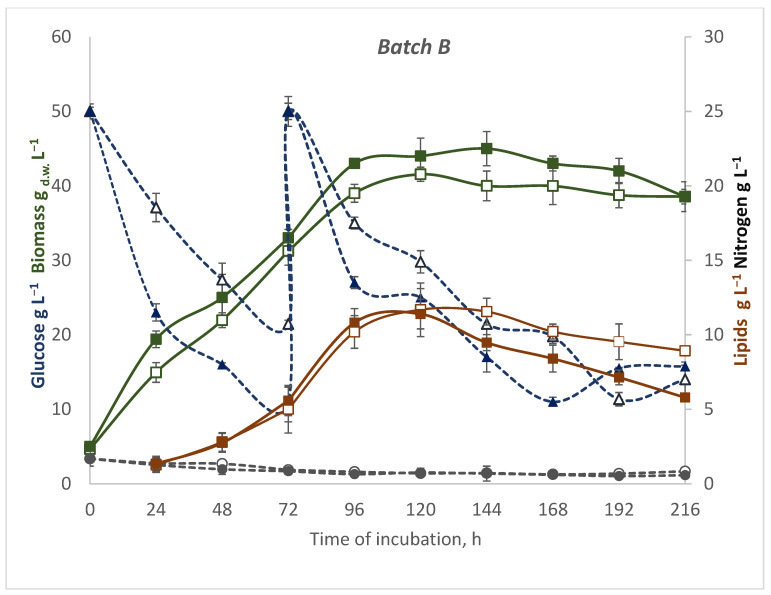
The timecourse of a fed-batch (B) culture of *R. glutinis* var. *rubescens* in DPW at two temperatures: 28 °C (filled tags) and 20 °C (empty tags). Kinetics of substrates: glucose—blue dotted line, nitrogen—grey dotted line, biomass (where _d.w._—dry weight)—green line, lipids—brown line. Values represent the average of duplicate experiments.

In the one-stage feeding (B) after the batch operation had run for 72 h, glucose was fed into the bioreactor to make 50 g of glucose per L. The biomass growth during the first 24 h after feeding (Figure 2) and the average value of biomass yields were 39 g L^−1^ at 20 °C and 43 g L^−1^ at 28 °C. These were correlated with glucose consumption (15 g L^−1^ and 23 g L^−1^, respectively) for 24 h after feeding. At the lower temperature, the maximal biomass yield (41.55 g L^−1^) was observed after 2 days from feeding (total time 120 h). Thereafter, the glucose consumption speed was lowered, and next 2 days, the biomass yield started to decrease. Regardless of the temperature, the one-step fed-batch strategy significantly resulted in an increased efficiency of lipid accumulation. The highest lipid content was observed after 144 h at 20 °C, and after 120 h under warmer conditions, respectively, it was 28.89 and 25.88 (% DW). Then, it started decreasing; however, lower temperatures allowed the cells a longer storage of accumulated lipid. The phenomenon where previously accumulated intracellular fat can be degraded has also been observed during *Rhodospordium toruloides* DSM 4444 cultivation on waste glycerol-based media with nitrogen remaining constant [43]. The authors’ suggestion was that yeast cells accumulate nonreserve lipid materials such as proteins or polysaccharides. Triglycerides stored in the lipid bodies of yeast are degraded via a cascade of hydrolysis reactions to DG, monoacylglycerols (MG), and FA. In our studies, we have not analyzed the content of other intracellular compounds. In future experiments, it will be necessary to control the number of living cells, and analyze the content of other components (proteins and polysaccharides) as well as the level and activity of endogenous lipases.

**Figure 3:**
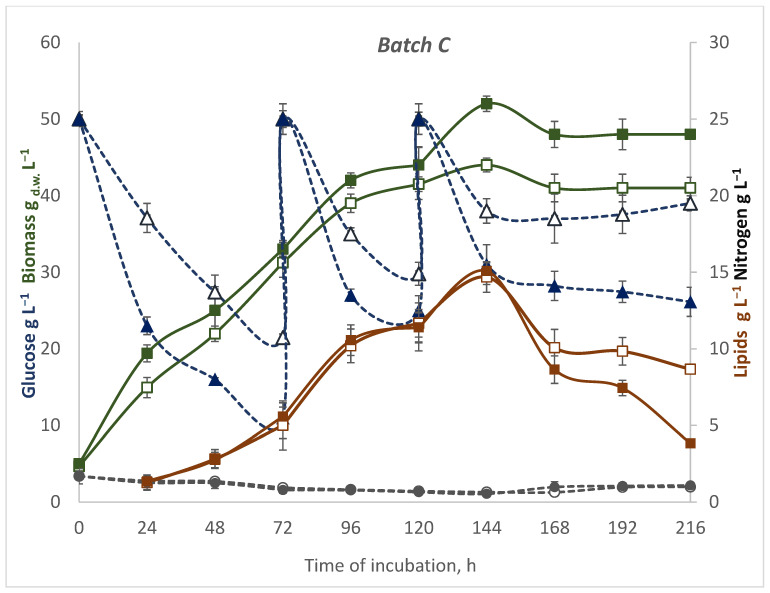
The timecourse of a fed-batch (C) culture of *R. glutinis* var. *rubescens* in DPW at two temperatures: 28 °C (filled tags) and 20 °C (empty tags). Kinetics of substrates: glucose—blue dotted line, nitrogen—grey dotted line, biomass (where _d.w._—dry weight)—green line, lipids—brown line. Values represent the average of duplicate experiments.

The two-stage pulse fed-batch (Figure 3) resulted in a high density of oleaginous cell cultures, and when yeast was culturing at 28 °C, the biomass yield achieved 52 g L^−1^ and 44 g L^−1^ at 20 °C. Under this condition, R*. glutinis* var*. rubescens* cells achieved a high yield of biomass. However, other strains were able to achieve much higher values of this parameter. The fed-batch process is suited to achieve high cell density. For example, after the cultivation of *Rhodotorula glutinis* CGMCC2258 at 24 °C in the bioreactor, the biomass reached 86.2 g/L [44]. During the fed-batch culture of *Saccharomyces cerevisiae*, a very high dried-cell-mass yield of 187.63 g/L was successfully attained by feeding a mixture of molasses and corn steep liquor [45]. Fed-batch cultivation modes have been broadly applied for microbial fat production, but only a few have been applied for *Rhodotorula* and glucose feeding [44,46,47]. Compared to these studies, the maximal biomass yield of *Rhodotorula glutinis* var*. rubescens* on DPW and glucose achieved in fed-batch C at 28 °C was lower, and the reason can be individual possibilities of the yeast strain, a different source of nitrogen, as well as incubation parameters.

The temperature of incubation also significantly influences glucose utilization. Only 12 g L^−1^ of glucose was utilized during 24 h of incubation at 20 °C after the second feeding, which was 24%. Under warmer conditions, yeast was able to utilize 38% of secondly added sugar, which does explain the higher biomass concentration. Meanwhile, the lipid content was increasing up to 29 (%DW). According to the high biomass yield, the volumetric lipid yield achieved the value of 15.08 g L^−1^. The lower temperature allowed for cells with the most efficient lipid accumulation, and on the sixth day of incubation, 34.15% of dry weight accounted for it. Eventually, the maximal volumetric lipid yield (14.68 g L^−1^) did not differ significantly, due to the temperature, because of the lower cell biomass at the same time. The next 24 h resulted in a drop (caused the decrease) in lipid concentration at both temperatures. The decrease in intracellular fat content was slower under the cooler condition. 

It is well known that the optimal conditions for the multiplication of cells do not always correspond to those that generate the highest production efficiency. An example is a high carbon-to-nitrogen ratio driving lipid accumulation in *Y. lipolytica* and limiting its growth. Quoting Papanikolau [48]: the concentration of ammonium ions in the growth media is considered to play a crucial role in redirecting the glucose flux toward metabolic pathways for the lipid accumulation and cell growth of oleaginous microorganisms. After nitrogen depletion, the oleaginous cells can continue consuming the glucose, which is converted into neutral lipids. Our study confirmed that after nitrogen depletion, rushing metabolism is directed to lipid biosynthesis (the fat content in the cells increases from 48 h when the nitrogen concentration is already low).

**Figure 4:**
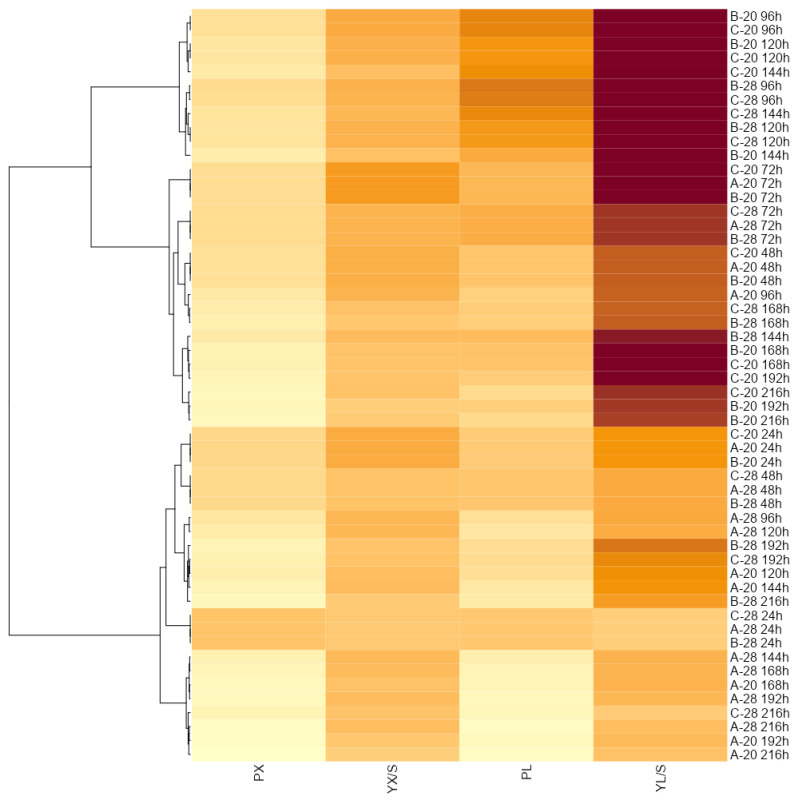
Heatmap based on productivity of biomass (P_X_), biomass/glucose yield coefficient (Y_X/S_), lipids productivity (P_L_), and lipid/glucose yield coefficient (Y_L/S_) at different batch types (A, B, C), temperatures of incubation (20–28 °C), and times of cultivation (24–216 h) (*p* < 0.05).

The heatmap (Figure 4) visualizes the differences in detail between biomass and lipids productivity (P_X_ and P_L_), biomass yield coefficient (Y_X/S_), and lipid yield coefficient (Y_L/S_), where the higher the color saturation, the higher the value of a given parameter. Generally, the analysis of the heatmap confirmed that the time of incubation is an important parameter in different comparative data. The fastest cell division and increase in biomass expressed by productivity occurred on the first cultivation day, and the highest value of Y_X/S_ was observed after 72 h at 20 °C in all types of batch incubation. The culture temperature did not differentiate the lipid productivity parameter in the first two days. The type of culture did not matter, because the conditions were identical at that time (except for the temperature). After the first feeding (72 h), noticeable differences were observed in both fat productivity and Y_L/S_. The latter parameter expresses how many grams of fat is produced from 1 g of consumed glucose [g g^−1^]. The further hours of cultivation (168–192 h) at a lower temperature confirmed that the yeast cells still consumed glucose for lipid biosynthesis, which was correlated with the productivity, especially after the second feeding.

The conversion yield of yeast biomass produced per glucose equivalent remained at very high values (max. Y_X/S_ = 0.931 at 20 °C, and 0.726 at 28 °C). *Rhodotorula glutinis* var*. rubescens* cells respire fully without secretion of fermentation byproducts such as a Crabtree-negative yeast [49], and this could affect the high growth rate, biomass yield, and Y_X/S_ value. The good examples supporting our hypothesis are the results of the growth of *S. cerevisiae* and *Issatchenkia orientalis* in molasses media during the batch-culturing. The average coefficient Y_X/S_ value after the triplicate experiments of *I. orientalis* Y116 (Crabtree-negative) was 0.807 g/g, when under the same condition of only 0.142 g/g for *S. cerevisiae* (Crabtree-positive) [50]. Furthermore, biomass yields higher than the theoretical maximal biomass yield of 0.51 (g g^−1^) were previously reported for *P. angusta, Y. lipolytica,* and *C. rugose* growing on glucose [51].

The higher-temperature incubation positively accelerated the process of yeast reproduction, wherein the highest biomass productivity was observed during the first two days of incubation. A longer acclimation to the lower temperature resulted significantly in the highest biomass yield coefficient value on the third day of incubation. 

At 28 °C, small differences in lipid productivity for batch C on the 4th and 5th day of incubation were observed. Both fed-batch and lower temperature increased the utilization of glucose toward lipid biosynthesis, as indicated by the Y_L/S_ values (Figure 4). The similarity is significant for 96, 120, and 144 h. The highest values of lipid production (14.68 g L^−1^), accumulation (34.15%, *w/w*), and volumetric productivity (P_L_: 0.1 g L^−1^ h^−1^) were reached by the strain in fed-batch C at 20 °C. Under this condition, the maximal lipid yield per unit of consumed glucose consequently received the highly satisfactory value of 0.242 g g^−1^, a value among the highest ones in the literature. 

Similarly, the biomass of *R. gracilis* ATCC 10788 was characterized by the highest intracellular lipid content (19 g/100 g _d.w._) after culture at 20 °C [52]. However, the intracellular lipid content as a function of different temperatures incubation was reversed for *Rhodotorula glutinis,* when the optimal 30 °C at day 6 resulted in 49.1% DW, 35.3 g/L, and 0.98 g/L/day, and the lower temperature of 25 °C decreased the lipid content significantly [53]. A similar correlation was also observed for *Yarrowia lipolytica* where a higher temperature of 33 °C led to reduced lipid yields [54]. However, 30 °C was the optimum temperature for cellular lipid accumulation (56%) by *Rhodotorula kratochvilovae* [55]. The response to low temperatures was studied on the *R. frigidialcoholis* strain previously isolated from ice-cemented permafrost soil in Antarctica [56]. There were cold-induced modifications to the cell membrane, as well as -induced RNAi mechanisms and switch metabolism. Compared to room temperature, at 0 °C, yeast cells overexpressed some genes, especially those involved in lipid metabolism and related to unsaturated fatty acids (FA) biosynthesis maintaining membrane fluidity. The other differences in transcript abundance were found in genes related to carbohydrate, glycan, and amino acid metabolism, as well as to signal transduction, translation, and transcription machinery. For other eukaryotic organisms such as copepod (*Paracyclopina nana*), low temperature increased the mRNA expression and the area of the lipid droplets [57]. The low temperature of incubation was beneficial for lipids biosynthesis by other yeast genera, such as *Metschnikowia* [4].

**Figure 5:**
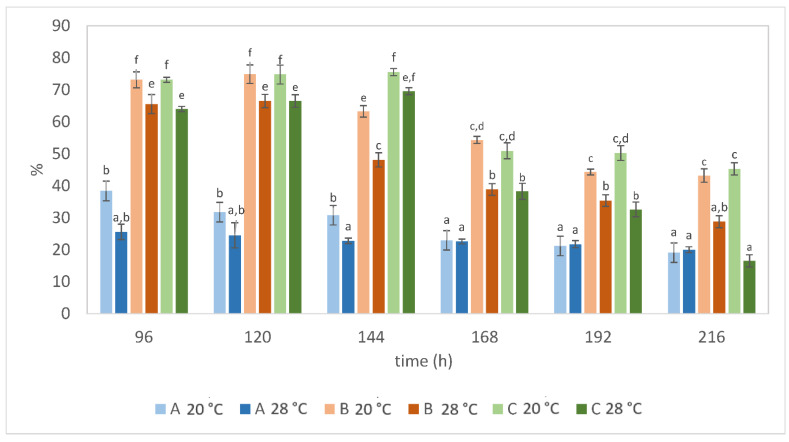
The efficiency of lipid production (ηL) as % of theoretical total lipid yield on the glucose (0.32 g g^−1^ according to Papanikolaou and Aggelis, 2011). ^a–f^ Values with different superscripts within a column are significantly different at *p*  <  0.05.

The theoretical total lipid yield on the glucose is 0.32 g g^−1^ [48]. We calculated the efficiency of lipid production proportionally to the theoretical total lipid yield as 100% (Figure 5). The glucose feeding strategy during the cultivation with potato waste had a significant effect on the efficiency of fat biosynthesis. The performance at the lower temperature was higher. It was noticed that lipids accumulated in the cells during the lower temperature remained in the cells for a longer time. Utilizing glucose and potato wastewater as a source of nitrogen, the strain achieved about 75.53% of the theoretical maximum yield (Batch C/20 °C). This indicates that there was still a little room for process improvement. Future studies should optimize other factors of fed-batch cultivation, which may include oxygenation/aeration ratio and agitation rate. Especially, fed-batch processes are important because the production rate and titers play a crucial role in its final cost [58]. This holds promise that the use of the waste starch for microbial lipid production will become economically feasible.

Oxygen, as the terminal electron acceptor in a respiratory cascade of aerobic microorganisms, participates in the formation of intracellular energy storage products such as carbohydrates and lipids [59]. The control of optimum dissolved oxygen (DO) is also essential to obtaining a high biomass yield during the fed-batch phase [60]. A high dissolved oxygen level at 50–60% enhanced the cell growth of *Trichsopron oleaginosus* [59]. The effects of the dissolved oxygen (DO) level on lipid accumulation were not examined in this study. The evolution of DOT (%, *v/v*) as a function of the culture time in shake-flask fermentations was discussed in depth by Papanikolaou et al. [61]. Nevertheless, it is worth mentioning that at the early steps of yeast cultivation, the dissolved oxygen tension value drops, remaining at >20% *v/v,* before increasing toward the end of the cultures [61]. The other authors found that 30% DO saturation provided the highest lipid accumulation in *Rhodosporidium azoricum*, *Y. lipolytica*, and *T. oleaginosus* [62,63]. A high final lipid content (51%) in *Trichosporon oleaginosus* was observed at low dissolved oxygen concentration (20–30%) during the lipogenesis phase [59]. There are some indications that low DO in the batch enhances the total lipids production of *Rhodotorula glutinis* [64].

The lipids of *R. glutinis* var. *rubescens* biomass (harvested after incubation at 28 °C) were characterized by a high content of oleic acid (^Δ9^C18:1) and palmitic acid (C16:0) (Table 2), and these two fatty acids accounted for over 69% of the total fatty acids. Under similar conditions (DPW and glucose, 28 °C) [8], a high content of linoleic acid, oleic acid, palmitic acid, and α-linolenic acid was observed in *T. domesticum* PCM 2960 lipids during incubation. The ability of cells to modulate membrane fluidity is one of the most important strategies of microorganisms for adaptation to low temperatures. An increased number of double bonds in fatty acid chains are beneficial to membrane fluidity at low temperatures, in addition to an elevated content of short-chain, branched-chain, and cyclic fatty acids [9,11]. In this study, lowering the culture temperature increased the oleic acid (over 72%) and decreased the palmitic acid biosynthesis efficiency. Overall, a significant increase in the sum of MUFA was observed when the temperature of incubation was 20 °C. The temperature of incubation did not influence significantly the PUFA content.

**Figure 6:**
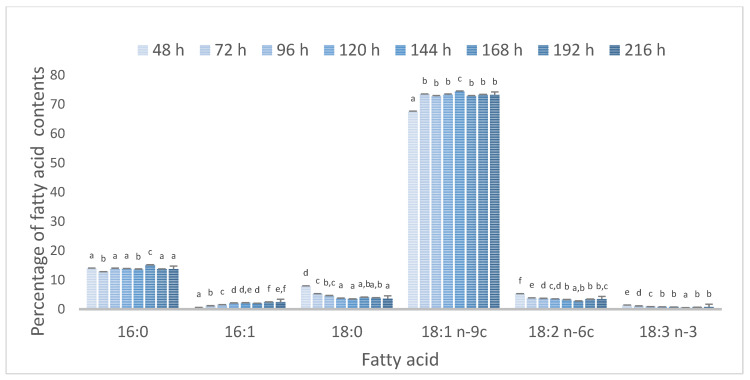
Dependence between main fatty acids composition and the time of incubation during fed-batch B at 20 °C. ^a–f^ Values with different superscripts for specific fatty acid within a column are significantly different at *p* < 0.05 (one-way ANOVA analysis).

Figure 6 shows the changes in the main fatty acids composition according to the time of incubation during fed-batch B at 20 °C. It should be noted that the greatest changes were observed after 24 h and the incubation time did not affect the composition of fatty acids. A similar tendency was observed regardless of the type of batch culture and temperature of cultivation. An approximate content of C18:1 and C16:0 was observed in the olive oil [65,66]. Lowering the culture temperature increased the linoleic acid biosynthesis efficiency during the cultivation of *Rhodotorula gracilis* ATCC 10788 [52], and the comparison of the composition of fatty acids with the composition of vegetable oils showed no similarity between yeast lipids and vegetable oils. However, compared to olive oil, the linoleic acid concentration was lower in *R. g.* var. *rubescens* lipids.

Double bonds, which are the structural basis of unsaturated acids, easily undergo oxidation processes, which consequently leads to unfavorable qualitative changes [67]. Secondary products of oxidation adversely affect the physical and sensory properties of the oil. A useful technique for fast and reliable determination of oxidative stability is calorimetry. Differential scanning calorimetry, DSC, describes the thermal transitions of examined samples on the micro-scale and can be used in many ways to provide information about crystallization and oxidative stability. In isothermal analysis, the oxidation induction time corresponds to the exothermic reaction between lipid and oxygen. The value of the induction time of an oxidation reaction is necessary to describe the stability of the oils and can be determined by the pressure differential scanning calorimetry (PDSC) method.

**Figure 7:**
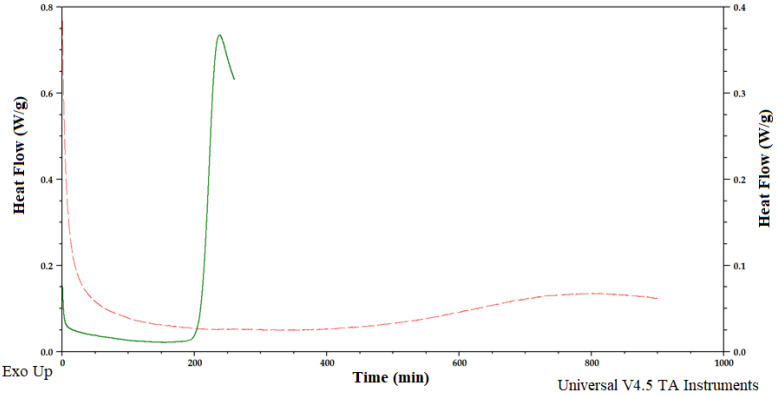
DSC curves of oxidation induction time of *Rhodotorula glutinis* var. *rubescens* lipid sample—dashed red line (Batch B, 20 °C, 120,) and olive oil—green line (as a control).

In this study, we examined yeast lipids from biomass after 120 h of incubation at all batch types. The example PDSC curve for extracted oil from *Rhodotorula glutinis* var. *rubescens* in the compartment to olive oil (control) is presented in Figure 7. It was observed that oxidation induction times (OITs) [68] of yeast oil averaged over 600 min, and the longest time of 770 min was observed for the yeast lipid sample after 120 h of batch B at 20 °C. Such values indicate the high oxidative stability of investigated oils. For comparison, under identical conditions, the induction time for olive oil was determined and was 238 min. The coconut oil or the liquid fraction obtained during fractionation of palm oil (palm olein) had the highest oxidative stability from important sources of edible oils and fats. 

The compounds that slow down the oxidation rate are known as antioxidants. Those substances, by scavenging free lipid alkyl radicals or lipid peroxy radicals, controlling transition metals, quenching singlet oxygen, and inactivating sensitizers [68], can extend the induction period. In the case of microorganisms, protecting cells against reactive forms of oxygen and radiation is the main role of carotenoids [69]. Growing as red colonies, *Rhodotorula* yeast can biosynthesize carotene, torularhodin and torulene pigments, which have antioxidant activity. It has been proven that osmotic stress and low temperature intensified the biosynthesis of β-carotene (up to 73.9% of the total carotenoid content) by *Rhodotorula toruloides* in media containing agro-industrial waste-potato wastewater [52]. Carotenoids as hydrophobics are conventionally extracted using organic solvents [70] and can therefore be transferred to the oils during the laboratory extraction process based on chloroform. It was observed in the different biological matrices [71] as microalgae [72,73] and red yeast [74]. That can explain the high oxidative stability of the studied lipids from *Rhodotorula glutinis* var*. rubescens* LOCKR13.

## 4. Conclusions

Cultivation in deproteinated potato wastewater fed with glucose enables the obtaining of a high yield of *Rhodotorula glutinis* var. *rubescens* LOCKR13 biomass (maximum 52 g _d.w._ L^−1^). The yeast strain showed different optimum temperatures for biomass and lipid production. The highest values of de novo lipid accumulation (34.15%, *w/w*), production (14.68 g L^−1^) and yield coefficients (Y_L/S_: 0.242 g g^−1^), and volumetric productivity (P_L_: 0.1 g L^−1^ h^−1^) were reached by the strain in fed-batch C (two-cycle) at 20 °C. The content of fatty acids depended mainly on the incubation temperature, and a significant increase in MUFA (especially oleic acid) was observed when the temperature of incubation was 20 °C. A unique set of thermal analyses of the microbial oil was performed. For the first time, it was experimentally proven that lipid extracted from red yeast has a high oxidative stability. This feature of the yeast oil can be useful for long shelf-life food products, and can be promising for the production of biodiesel.

## Figures and Tables

**Table 1:** Characteristics of deproteinized potato wastewater (DPW) used in this study (las vers) and its comparison to references.

COD	Dry Substance	TOC	Total Sugars	Directly Reducing Sugars	Total Nitrogen	Protein		P	K	Na	Ca	Mg	S	References
						Kiejdahl	Lowry							
gO_2_/L	g_d.w._/L	g/L	g/L	g/L	g/L	g/L	g/L	g/L	g/L	g/L	g/L	g/L	g/L	
30.0 ± 0.5	37.6 ± 0.0	14.7 ± 0.0	7.9 ± 0.1	7.0 ± 0.1	2.6 ± 0.0	16.0 ± 0.0	n.d.	0.42 ± 0.02	3.93 ± 0.25	0.58 ± 0.06	0.56 ± 0.04	0.310 ± 0.015	5.88 ± 0.93	[40]
35.12 ± 3.7	33.5 ± 0.5	n.d.	n.d.	13.8 ± 0.2	n.d.	12.9 ± 0.5	4.3 ± 0.4	n.d.	n.d.	n.d.	n.d.	n.d.	n.d.	[41]
n.d.	33.26 ± 0.43	n.d.	n.d.	4.40 ± 0.03	1.62 ± 0.06	n.d.	n.d.	0.33 ± 0.02	4.14 ± 0.32	n.d.	0.10 ± 0.01	0.24 ± 0.03	n.d.	[23]
30.82 ± 0.5	39.7 ± 0.5	n.d.	n.d.	7.3 ± 0.5	2.32 ± 0.07	14.5 ± 0.05	n.d.	0.27 ± 0.02	4.31 ± 0.25	0.06 ± 0.00	0.16 ± 0.01	0.24 ± 0.01	n.d.	[6]
n.d.	51.9 ± 0.3	22.9 ± 0.8	17.0 ± 0.1	16.4 ± 0.1	2.0 ± 0.1	12.5 ± 0.1	n.d.	0.29 ± 0.01	4.1 ± 0.4	0.73 ± 0.04	1.27 ± 0.2	0.27 ± 0.02	12.7 ± 2.1	[42]
28.9 ± 1.9	38.19 ± 7.4	11.72 ± 0.75	n.d.	4.49 ± 0.44	1.669 ± 0.047	10.43 ± 0.5	3.48 ± 0.27	0.66 ± 0.01	5.29 ± 0.90	n.d.	0.014 ± 0.002	0.261 ± 0.039	n.d.	This study

n.d.—not detected.

**Table 2:** The fatty acids range composition of extracted lipids from yeast biomass after 144 h of incubation.

Fatty Acid	Batch A	20 °C Batch B	Batch C	Batch A	28 °C Batch B	Batch C
C16:0	13.11 ± 0.59	13.52 ± 0.47	12.98 ± 0.39	17.83 ± 0.22	17.24 ± 0.17	17.56 ± 0.79
^Δ9^C16:1	2.41 ± 0.34	2.21 ± 0.14	2.5 ± 0.16	4.15 ± 0.19	3.85 ± 0.38	3.98 ± 0.21
C17:0	0.15 ± 0.03	0.11 ± 0.09	0.03 ± 0.00	2.80 ± 0.22	2.79 ± 0.15	2.81 ± 0.20
C18:0	4.26 ± 0.49	3.51 ± 0.52	5.02 ± 0.39	7.51 ± 0.34	7.49 ± 0.33	7.53 ± 0.22
^Δ9^C18:1	72.71 ± 1.66	74.37 ± 1.13	74.05 ± 1.59	50.25 ± 1.09	51.03 ± 0.98	51.52 ± 1.53
^Δ9,12^C18:2	3.69 ± 0.34	3.26 ± 0.39	3.53 ± 0.22	4.11 ± 0.45	4.17 ± 0.33	4.21 ± 0.59
^Δ9,12,15^C18:3	0.61 ± 0.16	0.74 ± 0.09	1.14 ± 0.55	1.32 ± 0.14	1.27 ± 0.32	1.24 ± 0.09
C22:0	n.d.	n.d.	n.d.	6.05 ± 0.39	5.85 ± 0.51	5.57 ± 0.09
Sum of others	1.44 ± 0.19	2.03 ± 0.29	1.57 ± 0.33	3.24 ± 0.24	3.14 ± 0.28	3.21 ± 0.21
∑SFA	18.43 ^b^	18.19 ^b^	18.68 ^b^	35.76 ^c^	34.92 ^c^	35.06 ^c^
∑MUFA	75.66 ^e^	77.45 ^e^	77.03 ^e^	54.72 ^d^	55.23 ^d^	55.85 ^d^
∑PUFA	4.30 ^a^	4.37 ^a^	5.08 ^a^	6.75 ^a^	6.68 ^a^	6.72 ^a^

∑SFA—the sum of saturated fatty acids, ∑MUFA—the sum of monounsaturated fatty acids, ∑PUFA—the sum of polyunsaturated fatty acids, n.d.—not detected. ^a–e^ Values with different superscripts are significantly different at *p*  <  0.05.

## Data Availability

Not applicable.
